# 1171. Follow-up of vaccine preventable disease hospitalisations in the ageing population: onset of chronic comorbidities

**DOI:** 10.1093/ofid/ofad500.1011

**Published:** 2023-11-27

**Authors:** Ahmed Salem, Maximilian Hartmann, Nathalie Servotte, Emmanuel Aris, T Mark Doherty, Ekkehard Beck

**Affiliations:** GSK, Wavre, Brabant Wallon, Belgium; Institute for Medical Information Processing, Biometry and Epidemiology, Munich, Bayern, Germany; GSK, Wavre, Brabant Wallon, Belgium; GSK, Wavre, Brabant Wallon, Belgium; GSK, Wavre, Brabant Wallon, Belgium; GSK, Wavre, Brabant Wallon, Belgium

## Abstract

**Background:**

Vaccine preventable diseases (VPD) such as influenza, pneumococcal infection, herpes zoster and pertussis pose a significant burden for adults 50 years and older. The disease burden beyond the acute phase (downstream effects) where a VPD can trigger or exacerbate comorbidities is less studied.

**Methods:**

Using Optum’s de-identified Clinformatics Data Mart Database, a retrospective claims database study was conducted to assess the diagnosis of new comorbidities in older adults (50+) up to 365 days following an index hospitalisation with a VPD during the epidemiological years 2016 – 2018. A subset of outcomes is reported here, focusing on the new onset of myocardial infarction (MI) and congestive heart failure (CHF). Subjects hospitalised with a VPD were compared to matched controls without a VPD hospitalisation. Matching was conducted on the day of VPD hospitalisation based on baseline variables like demographics, insurance, comorbidities, and Charlson-Comorbidity Index (CCI) score. The study analysed the new onset of MI and CHF over 365 days following a VPD index hospitalisation stratified by age category and CCI score at baseline. Results are reported as mean change (95% confidence interval (CI)).

**Results:**

At 365 days, there was a significant higher risk for a new MI observed in the VPD-hospitalised cohort compared to their matched controls (Figure 1) across all age groups and CCI categories. In particular, individuals without any comorbidity at baseline (CCI = 0) experienced an increased probability for a new MI diagnosis between 6% (4.9–7.4%, p< 0.001) and 13% (11.6% - 14.3%, p< 0.001). Furthermore, the VPD-hospitalised cohort also showed a significant higher risk for a new CHF diagnosis versus their non-VPD matched controls (Figure 2). Those without any comorbidity at baseline (CCI = 0) showed an increased proportion of new CHF diagnosis over 365 days between 12% (10–14%, p< 0.001) and 25% (24–27%, p< 0.001).

**Figure:**
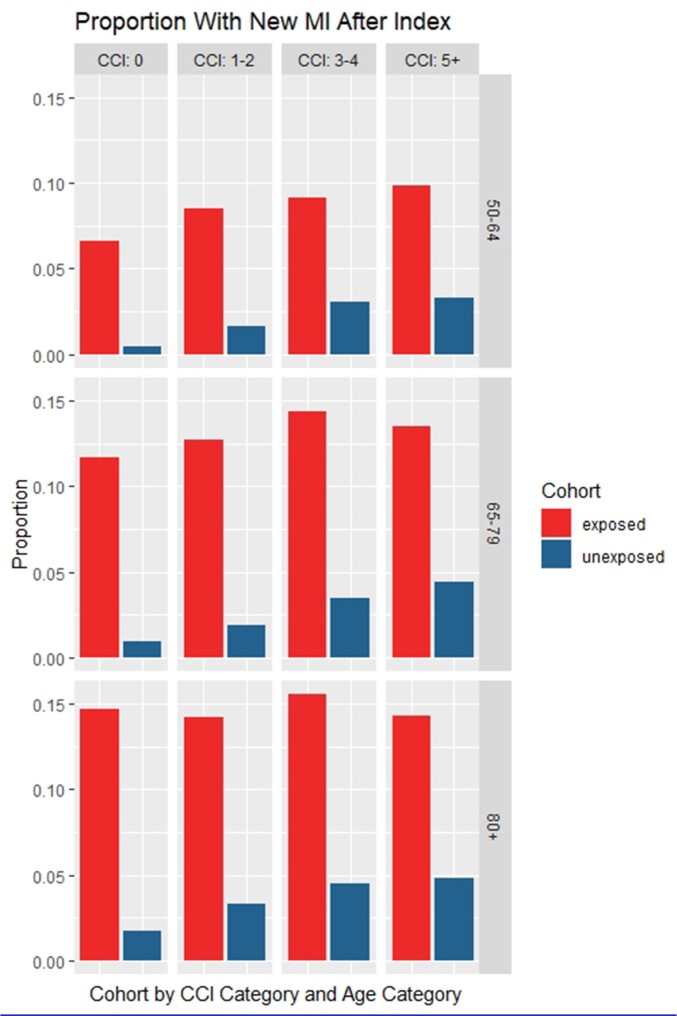

**Figure:**
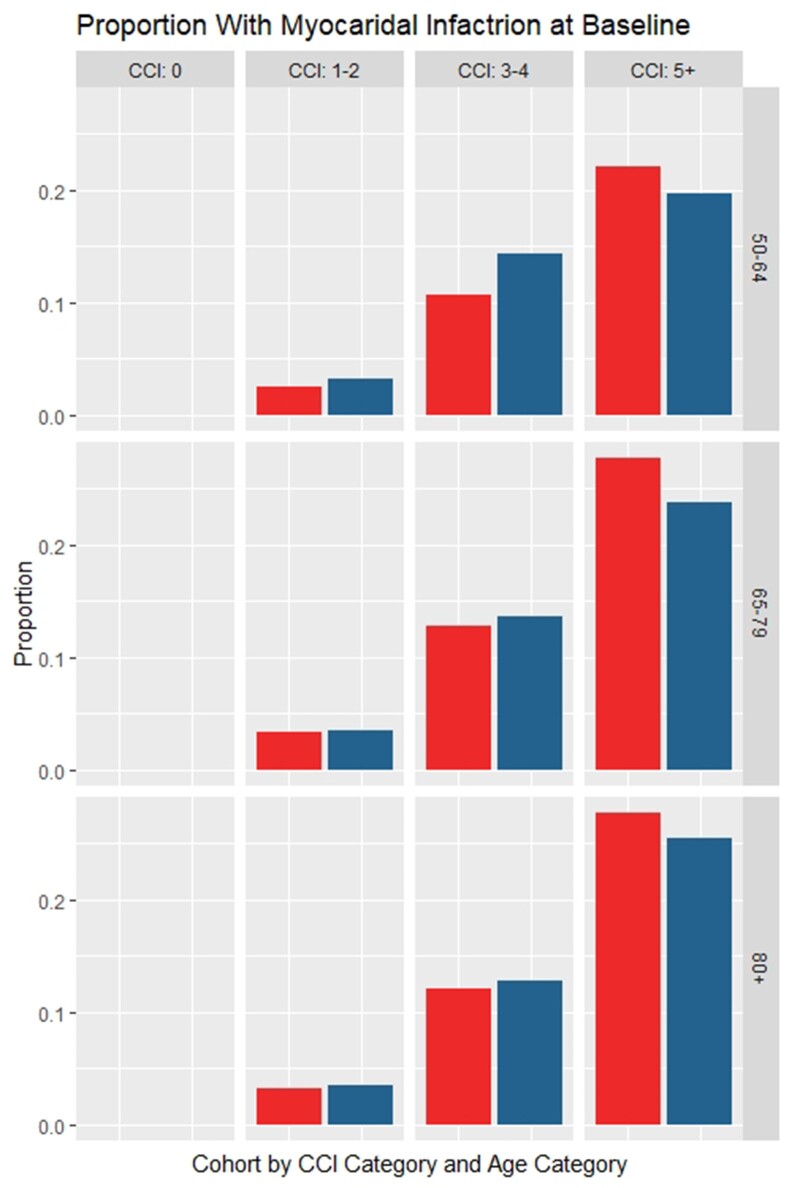

**Conclusion:**

Results suggest that individuals hospitalised for a VPD were more likely to experience a new MI or CHF compared to their matched controls. Limitations exist around direct associations between the VPD hospitalisation and the onset of a new MI or CHF diagnosis, but the findings support the hypothesis that downstream effects of VPDs could trigger or exacerbate a comorbidity.

**Disclosures:**

**Ahmed Salem, MSc**, GSK: employee|GSK: Stocks/Bonds **Maximilian Hartmann, PhD**, GSK: Grant/Research Support **Nathalie Servotte, PhD**, GSK: employee|GSK: Stocks/Bonds **Emmanuel Aris, PhD**, GSK: employee|GSK: Stocks/Bonds **T. Mark Doherty, PhD**, GSK: employee|GSK: Stocks/Bonds **Ekkehard Beck, PhD**, GSK: employee|GSK: Stocks/Bonds

